# The average relative dose intensity of R‐CHOP is an independent factor determining favorable overall survival in diffuse large B‐cell lymphoma patients

**DOI:** 10.1002/cam4.2008

**Published:** 2019-02-10

**Authors:** Monika Długosz‐Danecka, Sebastian Szmit, Tomasz Ogórka, Aleksander B. Skotnicki, Wojciech Jurczak

**Affiliations:** ^1^ Department of Haematology Jagiellonian University Krakow Poland; ^2^ Department of Pulmonary Circulation Thromboembolic Diseases and Cardiology Centre of Postgraduate Medical Education European Health Centre Otwock Poland

**Keywords:** average relative dose intensity, cardiotoxicity, chemotherapy, diffuse large B‐cell lymphoma, neutropenia

## Abstract

The prognosis of diffuse large B‐cell lymphoma (DLBCL) patients depends on lymphoma‐ and patient‐related risk factors and is best estimated by the international prognostic index (IPI). The aim of the study was to determine whether the average relative dose intensity (ARDI) of an anthracycline‐containing regimen could predict DLBCL outcome independently from the IPI. We analyzed 223 white Caucasian DLBCL patients who completed at least four cycles of first‐line immunochemotherapy with rituximab, doxorubicin, cyclophosphamide, vincristine, and prednisone (R‐CHOP). The ARDI was calculated by specially developed software in each individual patient, simultaneously with the chemotherapy prescription, which instantly revealed all causes of its decrease. The relevance of the ARDI for progression‐free/overall survival (PFS/OS) was evaluated. Prolonged intervals between cycles of immunochemotherapy—the most common cause of decreased ARDI (49.3%, 110/223)—were due to neutropenia (absolute neutrophil count <1.0 × 10^9^/L) and infections. Reductions in cytostatic doses were observed in 19.7% (44/223) of patients, mainly as the consequence of cardiotoxicity (23/223, 10.3%). The OS varied significantly when the ARDI was >90% (*P* < 0.00001). Multivariate analysis confirmed that an ARDI>90% was an IPI‐independent predictor of prolonged PFS (HR = 0.31; 95%CI: 0.20‐0.47; *P* < 0.00001) and OS (HR = 0.32; 95%CI: 0.21‐0.48; *P* < 0.00001). With an analytic tool allowing real‐time ARDI assessment, it was possible to maintain an ARDI above 90% in 161 of 223 patients (72%). DLBCL patients with an ARDI >90% have significantly better outcome regardless of the IPI; therefore, our official recommendation is an adequate dose density through efficient neutropenia prophylaxis and cardiac protection.

## 1 INTRODUCTION

The CHOP chemotherapy regimen, consisting of doxorubicin, cyclophosphamide, vincristine, and prednisone, remains the first‐line standard of care in diffuse large B‐cell lymphoma (DLBCL).^1^ Adding rituximab, an anti‐CD20 monoclonal antibody, was the only major modification thus far and has improved treatment efficacy.^2^ A correlation between the dose intensity and the therapeutic effect remains undefined.^3^, ^4^, ^5^, ^6^, ^7^

Dose intensity (DI) reflects the dose of the administered drug per unit of time (ie, expressed in mg/m^2^ per week). DI has been considered in the treatment of solid tumors, and recently, it was also considered in lymphoma therapy.^8^, ^9^ The relative dose intensity (RDI) expresses the amount of drug administered per unit of time compared to the planned amount of drug at the scheduled time. The intensity of the entire chemotherapy regimen is better defined by the average relative dose intensity (ARDI), which is a calculation of the mean values of the RDI of all drugs used in a chemotherapy cycle.

The optimal dose intensity of chemotherapy may be a specific challenge in aggressive lymphomas. Overall survival (OS) was significantly shorter when the RDI of doxorubicin and cyclophosphamide was below 80%.^8^ The effect of DI on the outcome of non‐Hodgkin's lymphoma patients was carefully evaluated for different chemotherapy regimens,^10^, ^11^ and the importance of an RDI of adriamycin >75% was also defined as the single most important predictor of survival in DLBCL.^9^ None of the mentioned trials have analyzed the effect of the ARDI in different international prognostic index (IPI) subgroups.

The aim of the current study was to determine whether the lymphoma treatment intensity expressed by the ARDI could be an IPI‐independent predictive and prognostic factor.

## 2 METHODS

### 2.1 Study cohort

The study group comprised 223 white, Caucasian, histopathologically confirmed treatment‐naive DLBCL patients who received immunochemotherapy including rituximab, doxorubicin, cyclophosphamide, vincristine, and prednisone (R‐CHOP) between 2005 and 2013. The IPI prognostic index was calculated for all patients at diagnosis.^12^ Efficacy and survival analyses were performed separately in low‐, intermediate‐ ,and high‐risk groups (with IPI: 0‐1, 2‐3, and 4‐5, respectively). The clinical stage of lymphoma was assessed by using the Ann Arbor classification with Cotswolds revision 1988.^13^, ^14^ The characteristics and demographics of patients are summarized in Table 1.

**Table 1:** Characteristics of patients in a study cohort: risk factor distribution and IPI analysis

Risk factor	Number of cases n (%)
Age	
≤60 y	133 (59,64)
>60 y	90 (40,36)
ECOG performance status	
<2	209 (93,72)
≥2	14 (6,28)
Clinical stage according to Ann Arbor scale	
I/II	73 (32,74)
III/IV	150 (67,26)
Number of extranodal sites	
0‐1	99 (44,39)
>1	124 (55,61)
Serum LDH activity	
N	97 (43,50)
>N	126 (56,50)
IPI	
0	19 (8,52)
1	47 (21,08)
2	70 (31,39)
3	50 (22,42)
4	34 (15,25)
5	3 (1,35)
IPI risk groups	
Low risk (L, IPI: 0‐1)	66 (29,60)
Intermediate risk (I, IPI: 2‐3)	120 (53,81)
High risk (H, IPI: 4‐5)	37 (16,59)

### 2.2 Oncological status, treatment, and dose intensity parameters

The ARDI was evaluated in a specially developed OWID^®^ computer program (dosage intensity assessment). The ARDI was calculated for all cycles of R‐CHOP immunochemotherapy based on the body surface area (BSA) of the patient, planned and actually administered doses of drugs, and planned and actual dates of chemotherapy cycles. The DI and RDI of each intravenously administered drug were assessed. R‐CHOP immunochemotherapy was to be repeated every 21 days for six cycles. None of the cases with fewer than four cycles was included, as including these cases would not allow a reliable assessment of the treatment DI.

All patients received supportive treatment, including prevention of tumor lysis syndrome, prophylactic antibacterial, antiviral and antifungal therapy, and transfusions of red blood cells, platelets, or other blood products, as required. Primary prophylaxis of neutropenia by granulocyte colony‐stimulating factor (G‐CSF) was not applied, and secondary prophylaxis was implemented according to local standards.

### 2.3 Response to treatment

Response to treatment was evaluated according to the Cheson criteria based on computed tomography (CT) and positron emission tomography (PET).^15^, ^16^ The progression‐free survival (PFS) time, which is defined as the time from the onset of R‐CHOP immunochemotherapy to lymphoma progression or death, was assessed. OS was calculated as the time from the beginning of treatment to death, regardless of the cause.

### 2.4 Statistical analysis

Kaplan‐Meier curves were used to determine PFS and OS. Univariate and multivariate analyses of the risk of lymphoma progression or death were carried out using the Cox proportional hazards model. The results were considered statistically significant if *P* < 0.05. All statistical analyses were performed using STATISTICA software.

## 3 RESULTS

**Figure 1:**
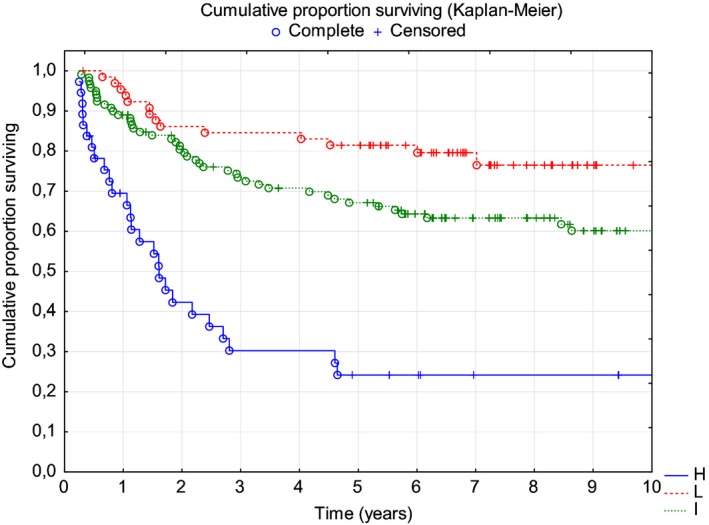
PFS according to the IPI (Kaplan‐Meier analysis, *P* < 0.00001)

**Figure 2:**
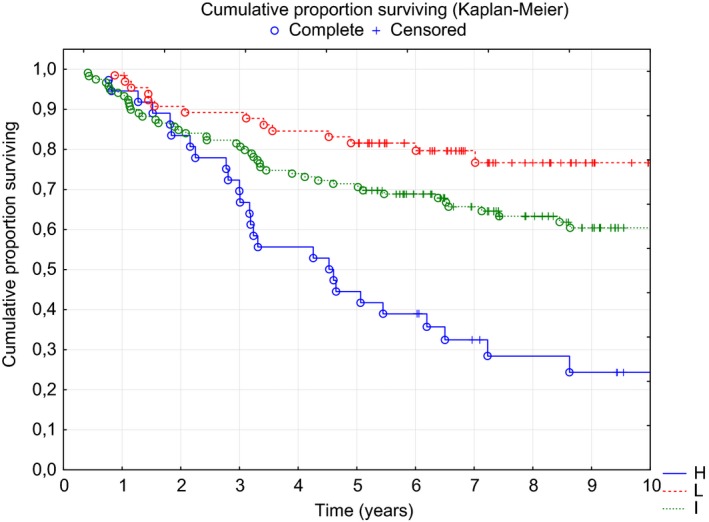
OS according to IPI (Kaplan‐Meier analysis, *P* < 0.00001)

In our group, the clinical characteristics and risk factor distribution were representative of DLBCL and comparable to those described in the literature (Table 1).^17^, ^18^ At the end of first‐line treatment, 150 (67.26%) patients achieved complete remission (CR), 62 (27.8%) partial remission (PR), 3 (1.3%) stable disease, and 7 (3.1%) progressive disease (PD). As anticipated, a high IPI (4 or 5) was associated with an increased risk of lymphoma progression and earlier death (median PFS and median OS 1.6 and 4.5 years, respectively), and patients with a low IPI (0‐1) had the best prognosis and had not reached the median PFS and OS at the median follow‐up of 6 years (Figures 1, 2). In our cohort, IPI risk factors that were most important for the prediction of progression were as follows: age over 60 years (HR = 1.73), elevated lactate dehydrogenase (LDH) activity (HR = 1.70), and extranodal location (HR = 2.14).

**Table:** 

IPI	High	Intermediate	Low
Median PFS	1.6 years	Not reached	Not reached

**Table:** 

IPI	High	Intermediate	Low
Median OS	4.5 years	Not reached	Not reached

**Figure 3:**
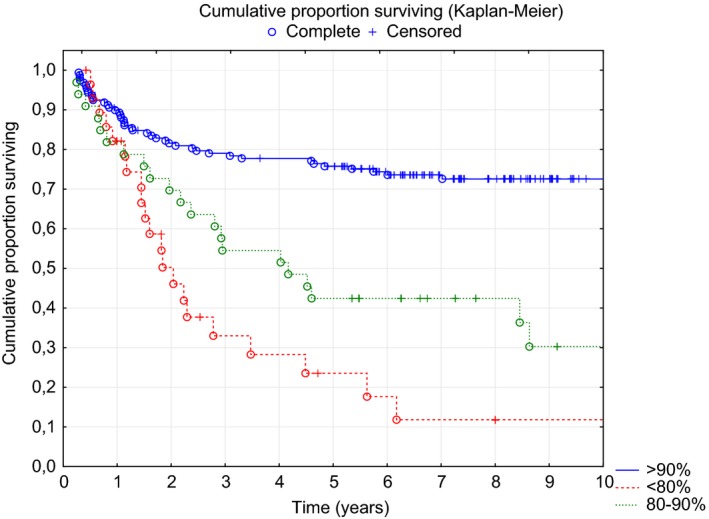
PFS according to the ARDI (Kaplan‐Meier analysis, *P* < 0.00001)

**Figure 4:**
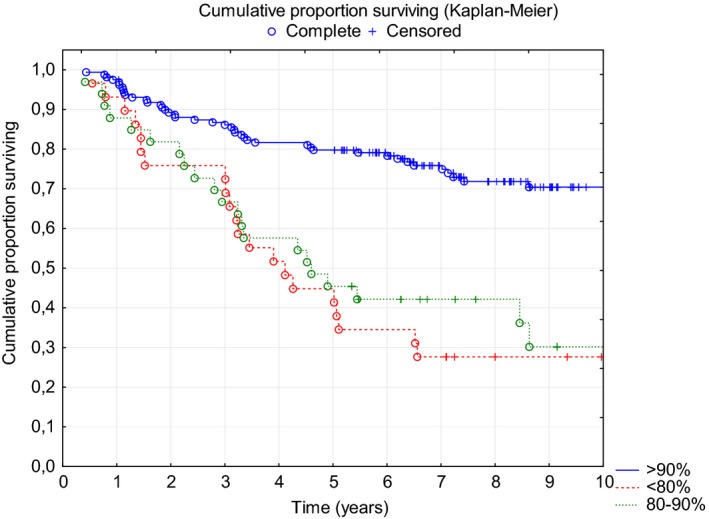
OS according to the ARDI (Kaplan‐Meier analysis, *P* < 0.00001)

Further analysis revealed that both PFS and OS depended on the ARDI of the R‐CHOP regimen. The median PFS was significantly different: 1.9 years, 4.1 years and not reached in patients with ARDI <80% (n = 29, 13%), 80%‐90% (n = 33, 14.8%), and >90% (n = 161, 72.2%), respectively (Figure 3). Surprisingly, Kaplan‐Meier curve analysis showed no significant difference in OS between patients with ARDI <80% and 80%‐90% (Figure 4), while the longest OS was observed in patients with ARDI>90%. In multivariate Cox proportional risk analysis, both a low IPI (0 or 1) and a high ARDI (>90%) during R‐CHOP immunochemotherapy were independent and favorable prognostic factors that were significant for predicting PFS and OS (Table 2).

**Table:** 

ARDI	<80%	80%‐90%	>90%
Median PFS	1.9 years	4.1 years	Not reached

**Table:** 

ARDI	<80%	80%‐90%	>90%
Median OS	4.0 years	4.6 years	Not reached

**Table 2:** Cox proportional risk model: ARDI >90% or low baseline IPI was correlated with favorable PFS and OS in a study cohort

Survival	ARDI >90%	Low IPI (0 or 1)
Univariate analysis		
PFS	HR = 0.28	HR = 0.39
	95%CI: 0.18‐0.44	95%CI: 0.22‐0.69
	*P* < 0.000001	*P* = 0.001
OS	HR = 0.30	HR = 0.43
	95%CI: 0.20‐0.46	95%CI: 0.24‐0.77
	*P* < 0.000001	*P* = 0.004
Multivariate analysis		
PFS	HR = 0.31	HR = 0.43
	95%CI: 0.20‐0.47	95%CI: 0.24‐0.76
	*P* < 0.000001	*P* = 0.004
OS	HR = 0.32	HR = 0.48
	95%CI: 0.21‐0.48	95%CI: 0.27‐0.85
	*P* < 0.000001	*P* = 0.01

HR, Hazard Ratio; CI, Confidence Interval.

The most frequent cause of a decreased ARDI is an extended time interval between R‐CHOP therapy cycles (110 of 223 patients, 49.3%), due to neutropenia and infections. Although an extended time between cycles did not exceed 7 days in the majority of cases (59 of 223, 26.5%), it was responsible for an over 10% decrease in the ARDI in 43 of 223 patients (19.28%).

In a cohort with an ARDI<80%, an even greater prolongation of the time intervals between cycles was observed: < 1 week in two patients (0.89%), 1‐3 weeks in five patients (2.2%), and above 3 weeks in 17 patients (7.6%). In an intermediate subgroup with an ARDI in the range of 80%‐90%, the interval time was extended to 1 week in 11 patients (4.9%), by almost 1‐3 weeks in nine patients (4%), and above 3 weeks in six patients (2.7%) patients.

The doses of anticancer drugs were reduced in 44 of 223 patients (19.7%); seven patients (3.1%) had reduced doses of ≥ 2 cytostatics (Table 3). The reduction in rituximab doses (18 patients, 8.1%) was mostly related to ampule dispensing and economic issues and not connected to adverse events; for this reason we did not consider this factor in the analysis of the causes of drug reduction. The most commonly reduced drug was doxorubicin (27 patients, 12.1%), mainly as a consequence of cardiotoxicity (23 patients, 10.3%). The doses of vincristine (in four patients; 1.8%) or cyclophosphamide (in two patients; 0.9%) were reduced due to neutropenia. For the same reason, all components of R‐CHOP were reduced in four patients (1.8%).

**Table 3:** Cytostatic dose reductions in a study cohort

	Total number of patients n (%)	ARDI <80 n (%)	ARDI 80‐90 n (%)	ARDI >90 n (%)
ADM	27 (12.11)	20 (8.97)	6 (2.70)	1 (0.45)
CTX	2 (0.89)	2 (0.89)	0 (0.00)	0 (0.00)
VCR	4 (1.79)	2 (0.89)	1 (0.45)	1 (0.45)
Rituximab	18 (8.07)	15 (6.73)	2 (0.9)	1 (0.45)

The causes of death in subgroups depending on the ARDI and IPI are presented in Table 4. The analysis identified 85 deaths (38.1%), 58 cases related to lymphoma progression, 20 cases of cardiovascular complications, and seven cases of death from other causes: multiorgan failure, secondary cancers, or infections.

**Table 4:** Cause of death in subgroups of the study cohort stratified by the ARDI or baseline IPI

		Deaths (all) n = 85	Lymphoma‐related n = 58	Cardiovascular n = 20	Other causes n = 7
IPI					
High	37 pts	26	22	2	2
Intermediate	120 pts	45	28	14	3
Low	66 pts	14	8	4	2
ARDI					
>90%	161 pts	43	29	12	2
80%‐90%	33 pts	21	14	5	2
<80%	29 pts	21	15	3	3

## 4 DISCUSSION

In lymphoma patients, the IPI (Table 1) remains clinically important^19^; however there have been many attempts to establish the potential prognostic role of chemotherapy DI and efficacy (8,9 10, 11). In DLBCL patients treated with R‐CHOP therapy, decreasing anthracycline RDI to <0.8 (80%) resulted in a significant decrease in complete responses (CR ‐ 52% vs 23%) and 5‐year OS (81% vs 54%).^8^ The 80% RDI threshold was also used in the study assessing DI in patients with breast cancer.^5^, ^6^ Several retrospective studies have shown that maintenance of the RDI of R‐CHOP can improve outcomes of patients with DLBCL, recognizing the RDI as an independent predictor of response and survival.^20^, ^21^ In another study, 157 DLBCL patients were prospectively evaluated, and R‐CHOP21 and R‐CHOP14 were shown to be equivalent regimens in terms of response and survival, but only if RDI reductions are avoided using clinical and support measures.^22^ Despite clear data, the dosage of cytostatics is often reduced, especially in older patients, to avoid treatment‐related side effects that would interrupt further therapy. However, the results of the retrospective study showed that older patients aged 70‐80 years who were treated with full treatment doses had better prognoses than patients with treatment attenuation; not all elderly patients are sufficiently healthy to tolerate the full‐dose treatment.^23^ The analysis of 479 de novo DLBCL patients aged 70‐79 years who were treated with R‐CHOP demonstrated that maintenance of the RDI was associated with improved outcome of elderly patients with DLBCL, suggesting that maintaining an RDI with adequate dose reduction is more important than uniformly administering a full dose of R‐CHOP to elderly patients.^24^

The role of G‐CSF in preventing neutropenia and infections was investigated in non‐Hodgkin's lymphoma patients (with the WHO classification, most of the participating patients would be currently classified as DLBCL).^25^ Neutropenia occurred in 37% of the G‐CSF‐treated patients and 85% of the controls, which indicates that the relative risk for control patients was 2.31 (*P* = 0.00001). A significantly greater DI was achieved in patients receiving G‐CSF without any additional drug toxicities. However, none of those observations led to the determination of the optimal ARDI level.

The aim of the current study was to determine whether the treatment intensity could be a prognostic factor independent from the IPI. In the multivariate analysis, we demonstrated that both a low IPI and an ARDI >90% may have independent positive predictive value for PFS and, consequently, OS. It has been shown that the probability of lymphoma relapse increases significantly with the decrease in the intensity of chemotherapy: the risk was highest when the ARDI was decreased to <80%, and the ARDI range of 80%‐90% was also unsatisfactory, especially for the prognosis of overall mortality. Previously, studies assessed the ARDI after treatment. In the Jagiellonian University Department of Haematology, the OWID^®^ computer program specially designed for dosage intensity assessment was introduced in 2007. This program allows us to check the average DI of the treatment during therapy, therefore helping us make optimal clinical decisions in real time.

As the extension of time intervals between R‐CHOP therapy cycles was the most common cause of a decreased ARDI (observed in 49% of patients). Improvement in the ARDI can be achieved by using G‐CSF in primary prophylaxis of neutropenia, in the prevention of infections and to better organize the ward routine to eliminate admission delays. The second most important problem is cardiotoxicity, which is the main reason for doxorubicin dose reduction and cardiac mortality. Our data (Table 4) confirmed the observations of a Polish Lymphoma Research Group (PLRG), showing that cardiac mortality is the second most common cause of death in lymphoma patients treated with R‐CHOP after the disease progression^26^ and that ensuring a good prognosis for patients with cardiovascular disorders is a special clinical challenge.^27^ Therapeutic strategies incorporating primary cardioprotection and close monitoring of cardiopulmonary capacity seem to be necessary. A number of potential cardioprotective therapies have been explored, and the most promising results were observed with the use of modified liposomal anthracycline,^28^ renin‐angiotensin system antagonists, and beta‐blockers 29, 30, 31; however, several combined cardioprotective measures may be more effective.^32^, ^33^

In most cases, a reduction in the rituximab dose results from rounding the amount administered to a full 100 mg, due to the size of the vials available and the relatively high cost of monoclonal antibodies. At present, due to the introduction of a central cytostatic dissolution laboratory in a hospital pharmacy, all doses are individually tailored. The need for dose reductions due to adverse reactions to rituximab was very rare, as prolonging infusion time and premedication with antihistamine drugs were adequate in most cases.

In conclusion, keeping the ARDI above 90% significantly increases OS in DLBCL patients receiving R‐CHOP therapy. It is therefore advisable to monitor the ARDI in all patients during therapy to allow the early introduction of primary neutropenia prophylaxis. The use of G‐CSF is widely accepted, but primary neutropenia prophylaxis should be further encouraged. Primary cardioprotection methods are not yet regarded as a standard of care. Implementing optimal cardioprotective strategies is particularly necessary in patients at increased risk of anthracycline cardiotoxicity, as the efficacy of R‐CHOP may be improved (by allowing a high ARDI) and the risk of cardiac mortality may be decreased.
